# Patient Safety and *Pro Re Nata* Prescription and Administration: A Systematic Review

**DOI:** 10.3390/pharmacy6030095

**Published:** 2018-08-29

**Authors:** Mojtaba Vaismoradi, Sara Amaniyan, Sue Jordan

**Affiliations:** 1 Faculty of Nursing and Health Sciences, Nord University, 8049 Bodø, Norway; 2 Faculty of Nursing and Midwifery, Tehran University of Medical Sciences, Tehran 1419733171, Iran; s-amaniyan@razi.tums.ac.ir; 3 College of Human and Health Sciences, Swansea University, Swansea SA2 8PP, UK; s.e.jordan@swansea.ac.uk

**Keywords:** PRN (*pro re nata*), medication systems, adverse effects, patient safety, nursing

## Abstract

PRN is the acronym for ‘*pro re nata*,’ written against prescriptions whose administration should be based on patients’ needs, rather than at set times. The aim of this systematic review was to explore safety issues and adverse events arising from PRN prescription and administration. Electronic databases including Scopus, PubMed [including Medline], Embase, Cinahl, Web of Science and ProQuest were systematically searched to retrieve articles published from 2005 to 2017. Selection criteria: we included all randomized controlled trials (RCTs) and studies with comparison groups, comparing PRN prescription and administration with scheduled administration, where safety issues and adverse events were reported. The authors independently assessed titles, abstracts and full-texts of retrieved studies based on inclusion criteria and risk of bias. Results were summarised narratively. The search identified 7699 articles. Title, abstract and full-text appraisals yielded 5 articles. The included studies were RCTs with one exception, a pre-test post-test experimental design. Patient populations, interventions and outcomes varied. Studies compared patient-controlled or routine administration with PRN and one trial assessed the effect of a practice guideline on implementation of PRN administration. More analgesia was administered in the patient-controlled than the PRN arms but pain reduction was similar. However, there was little difference in administration of psychotropic medicines. No differences between patient-controlled and PRN groups were reported for adverse events. The PRN practice guideline improved PRN patient education but non-documentation of PRN administration increased. This systematic review suggests that PRN safety issues and adverse events are an under-researched area of healthcare practice. Variations in the interventions, outcomes and clinical areas make it difficult to judge the overall quality of the evidence. Well-designed RCTs are needed to identify any safety issues and adverse events associated with PRN administration.

## 1. Introduction

PRN prescription and administration is a common practice [1]. PRN is an acronym for ‘*pro re nata*,’ authorising administration of medicine when needed, in the opinion of the nurse or patient administering medications, either at specified times of day or entirely at the nurse’s or patient’s discretion [2]. Most studies on PRN administration have concerned psychotropic medicines and investigated impact on symptoms such as sleep disturbances, emotions and psychoses in patients who have not achieved symptomatic and psychosocial recovery [3]. PRN regimens for disturbed behaviour and distress [4] or anxiety and agitation [5] are widespread in acute inpatient mental health settings. PRN prescription and administration of analgesia is also common after surgery [6].

As a feasible, patient-centred approach, PRN has the potential to encourage patients to participate in self-care [7] and manage signs and symptoms [8]. PRN prescription may increase efficiency of care [2]. The practice is widespread [9], with 68–83.9% of mental health patients receiving PRN-medication at least once during their care [5,10,11].

However, limited data are available on adverse events related to PRN administration [8] but the increased risks of harm due to PRN prescription and administration remains a concern [12,13]. No studies in the nursing literature has compared PRN administration in inpatient versus outpatient settings but there is a probable risk of non-adherence to medication regimen associated with inefficient monitoring by healthcare providers in outpatient settings. For instance, Miaskowski et al. [13] reported that in an oncology outpatient setting the patients’ adherence to their PRN analgesic regimen was only 22.2% to 26.6% during a 5-week period.

### 1.1. Description of the Intervention

Unscheduled medications fall into the categories of ‘*stat*’ and ‘PRN.’ *Stat* medication usually refers to prescription and administration of a one-off dose in addition to routine/regular medications prescription. PRN medication is prescribed in advance, with administration as-needed, according to clinical judgments or under instructions, written or verbal [12,14]. PRN prescription and administration creates an exceptional circumstance for patient care, allowing frequent or intermittent medicine use without direct physician supervision [7], typically involving analgesics, laxatives, sedatives, antiemetics, antipsychotics, anxiolytics and hypnotics [8,9,15]. PRN increases nurses’ involvement in decision-making and patient care, as it enables nurses to administer medication in a timely manner without having to call others to write new prescriptions [16]. The reasons for using PRN prescriptions should be continually monitored to avoid practice errors such as excessive doses, over-use and polypharmacy and ensure the efficacy of management plans [17]. During PRN prescription and administration, healthcare providers should record potential adverse drug reactions (ADRs) on a separate document in the patient’s medical file and share this with prescribers and pharmacists [18,19].

### 1.2. How the Intervention Might Work

PRN prescription gives healthcare providers latitude to administer medicines rapidly in acute situations or at the patient’s request [8,20]. If deployed appropriately, PRN administration improves treatment and relieves symptoms [2,21]. Conversely, abuse or misuse of PRN prescription and administration negatively influences patient care [2], for example by introducing polypharmacy, medication errors, adverse reactions, drug interactions and antipsychotic doses above recommended levels [22]. PRN prescription and administration has the potential to introduce dissonance between doctors and nurses [2].

### 1.3. Why It Is Important to Do This Systematic Review

While healthcare professionals agree that PRN prescription and administration is sometimes necessary for high quality patient care [2], the evaluation of the efficacy and effectiveness of PRN description and administration is difficult, because PRN administration relies on healthcare providers’ perceptions [10] and their interpretations of prescribers’ intentions [23]. Some interventions have been suggested to improve PRN prescription and administration, such as separate medication administration records for PRN and educational programs for healthcare providers [1]. A previous review indicates that there are few studies to support PRN prescription [24] and current practice is based on clinical experience and habit rather than high quality evidence [20]. This systematic review offers a background on PRN prescription, focusing on safety issues.

## 2. Aim

The aim of this systematic review was to investigate patient safety and adverse events arising in conjunction with PRN prescription and administration across healthcare settings.

## 3. Materials and Methods

### 3.1. Criteria for Considering Studies for This Systematic Review

We included studies on the prescription and administration of PRN to patients receiving nursing care. Studies with an emphasis on the efficacy and safety of PRN compared with other types of medication prescription and administration were included.

### 3.2. Types of Studies

We sought all relevant randomized controlled trials (RCTs). Due to the low numbers of RCTs of PRN, we included trials from diverse settings.

### 3.3. Types of Participants

Any hospital or care home inpatients or outpatients that received PRN prescription and administration by healthcare providers were considered.

### 3.4. Types of Interventions

○ Any short or long-term medication prescription with administration at the discretion of healthcare providers (PRN) was considered.
○ The ‘as prescribed’ pattern of prescription and administration compared with the PRN pattern.

### 3.5. Types of Outcome Measures

Only studies reporting adverse events or ‘patient safety’ were included. We considered a range of outcome measures including patient-reported outcomes and process outcomes.

### 3.6. Search Methods for Identification of Studies

Electronic databases including Scopus, PubMed [including Medline], Embase, Cinahl, Web of Science and ProQuest were systematically searched to retrieve articles published between 2005 and 2017, without language restrictions. The search strategy consisted of the keywords below, based on the authors’ experiences and controlled vocabularies such as the MeSH (medical subject headings):

“Drug-Related Side Effects and Adverse Reactions” or “Adverse Drug Event” or “Adverse Drug Reaction” or “Drug Side Effects” or “Drug Toxicity” or “Side Effects of Drugs” or “Toxicity, Drug” and “PRN (*pro re nata*)” or “as needed” or “as required.”

“Drug-Related Side Effects and Adverse Reactions” or “Adverse Drug Event” or “Adverse Drug Reaction” or “Drug Side Effects” or “Drug Toxicity” or “Side Effects of Drugs” or “Toxicity, Drug” and “PRN (*pro re nata*)” or “as needed” or “as required” and Nurs*.

References in the reviewed articles were backtracked. The indices of well-known journals publishing in this area were searched.

### 3.7. Data Collection and Analysis

#### 3.7.1. Selection of Studies

Three authors (M.V., S.A. and S.J.) independently screened titles and abstracts from the retrieved articles and decided which studies met the inclusion criteria: peer-reviewed RCTs in caring sciences, focus on PRN and published in online scientific journals. Next, two independent review authors (M.V. and S.J.) assessed the full-text of selected articles to ensure that they met the above-mentioned inclusion criteria using the methodological checklist developed by National Institute for Health and Care Excellence (NICE) [25]. In case of disagreements, discussions were held to reach consensus.

#### 3.7.2. Data Extraction and Management

Two review authors (M.V. and S.A.) independently extracted the details of articles included in the review in terms of design, sample, intervention, prescription and administration and outcome measurement.

#### 3.7.3. Assessment of Bias in Included Studies

Risk of bias is any error or deviation in the design, study process, analysis and reporting of RCTs, which can cause an underestimation or overestimation of results or inferences [26]. Two authors (M.V. and S.J.) assessed each selected article using the Cochrane Collaboration’s risk of bias tool [26]. This comprised: ‘selection bias,’ including random sequence generation, allocation concealment; ‘performance bias,’ including blinding of participants and personnel; ‘detection bias,’ including blinding of outcome assessment and incomplete outcome data assessments; ‘reporting bias,’ including selective reporting; and ‘other bias,’ such as conflict of interests.

#### 3.7.4. Measures of Treatment Effect and Unit of Analysis

The heterogeneity of the articles precluded a meta-analysis. Results are presented narratively.

#### 3.7.5. Dealing with Missing Data and Assessment of Heterogeneity

Since a meta-analysis could not be performed, no articles were excluded due to missing data and there was no assessment of heterogeneity.

#### 3.7.6. Data Synthesis

We used a theoretical framework of patient safety to accommodate the studies’ heterogeneity in terms of designs, participants and interventions.

#### 3.7.7. Quality of the Evidence

The authors employed the ‘grading of recommendations assessment, development and evaluation’ (GRADE) criteria [27,28] to assess the quality of the articles.

#### 3.7.8. Subgroup Analysis, Investigation of Heterogeneity and Sensitivity Analysis

These could not be undertaken, due to inability to pool results.

## 4. Results

### 4.1. Description of Studies

Although no language limitations were applied, all relevant articles were in English. Five articles on the safety and efficacy of PRN prescription and administration are included in this systematic review. The characteristics of the studies are presented in Table 1.

### 4.2. Results of the Search

**Figure 1:**
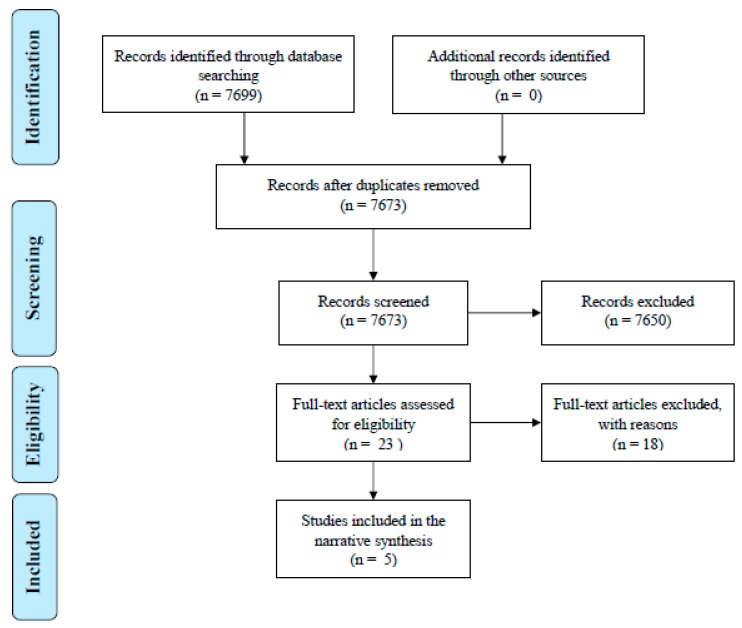
Study flow diagram according to the PRISMA.

The search identified 7699 articles that could be potentially included in the review. From independent appraisal of the titles and abstracts of the articles by two authors (M.V. and S.J.) deleting duplicates (26 articles) and articles not meeting the inclusion criteria (7650 articles) led to the selection of 23 articles. Reading the full-text of the articles by two authors of this systematic review (M.V. and S.J.) for the inclusion criteria and the selection of RCTs over other study designs led to inclusion of 5 articles. Manual search in the references lists of the included studies identified no more articles. The process of the search is described using the preferred reporting items for systematic reviews and meta-analyses (PRISMA) flowchart in Figure 1.

### 4.3. Included Studies

The included studies (n = 5) were published between 2005 and 2015. Two studies [29,30] were conducted in the USA, one [15] in the UK, one [32] in South Korea and one [31] in Iran. All studies were small with 25–161 participants recruited.

### 4.4. Design

Three studies [30,31,32] were parallel group RCTs. Chibnall et al. [29] was a cross-over RCT. Baker et al. [15] used a pre-post exploratory design.

### 4.5. Interventions

Interventions varied. Three studies considered analgesia [29,30,31], one a phosphodiesterase inhibitor (udenafil) [32] and one a practice manual [15] (Table 1).

### 4.6. Outcomes

The outcomes in the selected articles were diverse:

#### 4.6.1. Psychological Health

Only Chibnall et al. [29] measured behaviour and emotional wellbeing as the primary outcome and agitation as a secondary outcome.

#### 4.6.2. Appropriateness of Prescription and Administration

In Chibnall et al. [29], routine and PRN psychotropic medication use recorded in nursing home records was a secondary outcome. Baker et al. [15] evaluated the prescription and administration of PRN psychotropic medicines by weekly audits of nursing notes and prescription records; consenting nursing staff were asked why PRN psychotropics were administered and staff were asked to evaluate the practice manual by postal questionnaire. Morad et al. [30] assessed analgesic administration records following each pain assessment. Hajimaghsoudi et al. [31] assessed the adherence to the dosing schedule based on the number of returned tablets.

#### 4.6.3. Physical Health

Morad et al. [30] measured pain, analgesic use, sedation, vital signs (respiration rate, oxygen saturation, heart rate, systolic blood pressure). Patients were monitored continuously hourly in the first 10 h and then every two hours until discharge from the ward or the collection of 16 h of data. Neurological deterioration was also assessed. Hajimaghsoudi et al. [31] assessed ankle pain and swelling at rest and full weight bearing at baseline and follow up at day 7 as the primary outcome. Park et al. [32] gave a primary efficacy end point as changes in a subscore of the International Index of Erectile Dysfunction questionnaire; vascular endothelial markers and vital signs were assessed before and after treatment.

#### 4.6.4. Adverse Events

Diverse adverse events were monitored, including:

- The adverse effects of paracetamol [29],
- Medication errors associated with PRN prescription and administration [15],
- Neurologic deterioration, excessive sedation, nausea, vomiting, pruritus, insufficient analgesia, and/or respiratory insufficiency [30],
- The adverse effects of the medicines, such as gastrointestinal bleeding or upset, as a secondary outcome [31],
- Safety and ADRs using twelve-lead electrocardiograms at screening, after 8 weeks’ treatment and during the treatment-free follow up period [32].

#### 4.6.5. Excluded Studies

Eighteen studies were excluded [16,21,23,24,33,34,35,36,37,38,39,40,41,42,43,44,45,46]. Details of the excluded studies are in Table 2.

#### 4.6.6. Risk of Bias in Included Studies

The risk of bias varied between studies (Table 3).

#### 4.6.7. Allocation

There were variations in the processes of random sequence generation and concealment among the studies (Table 1).

#### 4.6.8. Blinding

Chibnall et al. [29] was blinded. Hajimaghsoudi et al. [31] stated that blinding of participants was impossible but investigators were blinded. Park et al. [32] was open-label. Other studies did not report blinding [15,30].

#### 4.6.9. Incomplete Outcome Data

Attrition is reported in Table 3. Incomplete data documentation might have led to high risk of attrition bias in the studies by Baker et al. [15] and Hajimaghsoudi et al. [31].

#### 4.6.10. Selective Reporting

All studies followed their protocols and reported their findings accordingly.

#### 4.6.11. Other Sources of Bias

Baseline characteristics of participants were similar in all studies. Cross-over design may have minimized the risk of allocation bias in the study of Chibnall et al. [29].

#### 4.6.12. Effects of Interventions

##### Psychological Health Outcomes

Chibnall et al. [29] found that during the intervention phase, patients spent more time in media engagement (*p* = 0.01), direct social interactions (*p* = 0.05) and work-like activity (*p* = 0.06) than during the placebo phase. They spent less time during the treatment phase engaged in independent self-care (*p* = 0.02). Emotional wellbeing, agitation, sleeping and independent walking did not differ between study phases (*p* = 0.80). No other studies reported psychological outcomes.

##### Prescription and Administration of Medicines

In the cross-over trial by Chibnall et al. [29], the presence/absence of psychotropic medication did not vary with routine use of acetaminophen.

Baker et al. [15] investigated the impact of a manual on prescription and administration of psychotropics. Over 10 weeks, the patients received 484 doses of psychotropics PRN. Three patients received more than 50 doses and 7 patients did not receive any PRN medications. The types of drugs changed significantly during the study: benzodiazepines and antipsychotics were reduced but z-drugs (zopiclone) increased. Many drugs were administered on their own but 12 different combinations of drugs were used, mainly haloperidol plus lorazepam. 36.5% of prescribed maximum PRN doses of antipsychotics were equal to or above the British National Formulary advisory limits. The quality of nursing notes fell and the non-documentation of PRN administration increased after the introduction of the manual. There was no documented evidence of side-effect monitoring for any dose of PRN administered during the study in either arm. The mean prescription quality assessed by separate eight-point quality rating scales increased but this was not statistically significant. The provision of information and education to patients recorded on forms provided increased significantly after manual introduction. Staff found the manual well-organized, helpful and understandable.

Morad et al. [30] explored post-operative analgesia. Nurses permitted patients in the PCA group to receive IV analgesic therapy for longer periods of time than those in the PRN group. For a given level of pain, the PCA group used almost twice as much fentanyl as the PRN group, with considerable inter-patient variability.

Hajimaghsoudi et al. [31] explored naproxen for ankle sprain bd versus PRN. More tablets were returned unused in the PRN group.

##### Physical Health Outcomes

Morad et al. [30] found patients in the IV PCA group reported less rest pain but received more fentanyl, than patients in the PRN group (3.7 vs. 5.2 and *p* = 0.003 and 54.8 vs. 29.9 g/h and *p* = 0.002).

Hajimaghsoudi et al. [31] reported that overall pain reduction on weight bearing and at rest was not significantly different between arms.

Park et al. [32] reported that erectile function was improved in the once-daily and PRN arms after 8 weeks of treatment. No significant differences between the groups were found; mean values for all biomarkers at baseline and after 8 weeks’ treatment were within normal ranges in both arms.

##### Adverse Events and Errors

Chibnall et al. [29] reported that 2 out of 25 patients experienced serious adverse effects not attributed to study medication (paracetamol) including collapse due to cardiac ischemia and hip fracture.

Baker et al. [15] reported medication errors, excluding poor quality prescribing, in 23 of 35 patients. Errors included: failure to stop PRN when a regular dose had been prescribed—one patient received a 30mg daily dose of olanzapine; same medicine prescribed twice as PRN; omitting to cross off prescriptions, leading to administration of the same drug from two identical prescriptions—one patient received two different antipsychotics regularly and was prescribed a further two as PRN. Administration errors included: administration of doses other than that prescribed (usually lower), inconsistent documentation, inconsistencies between the treatment protocol and nursing notes.

Morad et al. [30] reported no differences between PCA and PRN groups in terms of sedation, coma, respiratory rate, systolic blood pressure, heart rate, nausea, vomiting, oxygen saturation or neurological deterioration.

Hajimaghsoudi et al. [31] found the PRN regimen was safer than the twice daily regime with lower total drug doses. No serious adverse events were reported.

Park et al. [32] reported that udenafil was well-tolerated in both once-daily and PRN arms and most adverse events were mild to moderate. The most commonly reported treatment-related ADRs were flushing and headache. No significant differences were found between arms with regard to treatment-related ADRs or biomarkers of endothelial function.

## 5. Discussion

### 5.1. Summary of Main Results of This Review

The authors aimed to investigate safety issues and adverse events associated with PRN (*pro re nata*) prescription and administration in healthcare settings. Few randomized controlled trials compare PRN medication regimens with regular administration of the same drug [20]. We identified only four such trials addressing safety and adverse events. Variations in the findings in the current review allow no firm conclusions but the frequency and duration of analgesic use were higher with PCA than PRN administration [30]. Pain reduction was similar with routine and PRN prescriptions [31,32]. The introduction of a PRN practice manual reduced antipsychotic prescriptions, as patients were switched to hypnotics and reporting of patient education increased but nursing notes deteriorated and reporting of adverse effects remained zero [15].

### 5.2. Overall Completeness and Applicability of Evidence

The paucity and size of relevant studies, diversity of designs, variations in populations and multiple interventions highlight the incompleteness of the evidence in this systematic review on patient safety and adverse events related to PRN administration. More studies are needed to explore whether PRN prescriptions and the associated transfer of decision-making to nurses or patients and reduced bureaucracy, affects patients’ well-being and quality of care. The safety of PRN prescriptions may depend on appropriate education for nurses [23,38,44,46] or patients [39] and new technologies might improve access to information but we found little evidence for this.

### 5.3. Quality of the Evidence

Low sample sizes, difficulties with blinding, absence of information on sampling, randomization and attrition in some trials, variations in the designs, interventions, outcomes and results suggest that the overall quality of evidence is very low. The issues affecting the quality of the included studies differed and mainly stemmed from a lack of detail regarding the methods and interventions in the individual studies. Detailed reporting of the signs and symptoms of ADRs or ‘undesirable effects’ as listed in manufacturers’ literature [47] is essential to improve the work on the effectiveness of PRN medication regimens. No studies monitored patients for these safety issues, detracting from the data and the quality of the research.

### 5.4. Potential Biases in the Review Process

We tried to reduce bias during this review by conducting a thorough literature search using different keywords and databases. The Cochrane Risk of Bias Assessment is provided in Table 3.

### 5.5. Agreements and Disagreements with Other Studies or Reviews

A previous systematic review [20] of PRN medication regimens for seriously ill people in hospital reported that no evidence from RCTs supporting PRN administration and current practice is based on clinical experience rather than evidence. No further reviews were identified for comparison with our findings.

## 6. Conclusions

### 6.1. Implications for Practice

Insufficient evidence for PRN administration and prescription suggests that PRN safety issues and adverse events are under-recognized. The development and implementation of PRN guidelines described in one of the studies [15] did little to modify errors or improve clinical outcomes but might be useful to improve patient education. Nurse managers and policy makers need to establish educational programs for improving healthcare providers’ knowledge of PRN prescription and administration, how to monitor it and report ADRs and related safety issues [47].

### 6.2. Implications for Research

Well-designed RCTs of PRN prescription and administration are needed to explore patient safety. The efficacy and effectiveness of PRN with other methods of medication administration and prescription is under-explored but our diverse findings suggest that safety will depend on context, both clinical area and staff preparation. PRN practice guidelines should be developed and evaluated [21], with a focus on ADR monitoring, reporting and analysing safety data to increase confidence in this crucial but under-researched practice.

## Figures and Tables

**Table 1:** Summary for the comparisons of findings between the studies.

Title	Year of Publication	Country	Aim	Participants	Methods	Findings for the Main Comparison *		
						Outcome	Impact	Agreement between Studies
Effect of acetaminophen on behaviour, well-being and psychotropic medication use in nursing home residents with moderate-to-severe dementia [29]	2005	USA	To evaluate the effect of regularly scheduled administration of analgesic medication compared with analgesia ‘as needed’ on behaviour, emotional well-being and use of as-needed psychotropic medications in nursing home residents with moderate-to-severe dementia.	25 nursing home residents in two nursing homes, 3 male and 22 females, with a mean age of 85.9 ± 7.4	Randomized, double-blind, placebo-controlled, crossover trial.	Psychological effects	No effect was reported on emotional well-being, agitation or quality of life.	High
						Appropriateness of prescription and administration	The frequency of psychotropic use by routine and PRN administration did not differ between study arms and phases.	High to moderate
						Adverse events	Some adverse events unrelated to the medication and drug use pattern were reported.	High to moderate
The impact of a good practice manual on professional practice associated with psychotropic PRN in acute mental health wards: an exploratory study [15]	2008	UK	To assess the effect and acceptability of a good practice manual on prescribing and administration practices of PRN psychotropic medication in acute mental health wards.	12 physicians, 11 nurses and 35 patients in two acute mental health wards; gender and age of participants were not reported.	Pre-post exploratory design. Methods of sampling and patient selection were not reported	Appropriateness of prescription and administration	The practice manual influenced the administration and prescription of PRN medication. The prescription and administration of benzodiazepines and antipsychotics were reduced, whilst the z-drugs were increased. The maximum doses of antipsychotics administered using PRN prescriptions were above safety limits stated in the British National Formulary. Patient education and provision of information were increased. Inappropriate or excessive doses, duplicated co-prescriptions, administration errors and problematic documentation were reported in both arms.	High to moderate
The efficacy of intravenous patient-controlled analgesia after intracranial surgery of the posterior fossa: a prospective, randomized controlled trial [30]	2012	USA	To investigate whether IV patient-controlled analgesia (PCA) would lead to reductions in postoperative pain after neurosurgical procedures involving the posterior fossa compared with conventional IV nurse-administered as-needed (PRN) therapy.	80 patients undergoing elective surgery in the neurology critical care unit. The male/female distributions were 31/21 and 11/20 and the mean ages were 41.4 ± 11.1 years and 45.4 ± 14.6 years for two study arms.	Block randomized controlled trial	Appropriateness of prescription and administration	Patients in the PCA arm received more analgesia and had more pain relief than the PRN arm	High to moderate
						Physical effects	Patients in the PCA group reported less severe pain than the PRN group.	High
						Adverse events	Some adverse events unrelated to the medication and drug use pattern were reported.	High to moderate
Naproxen twice daily versus as needed (PRN) dosing: efficacy and tolerability for treatment of acute ankle sprain, a randomized clinical trial [31]	2013	Iran	To compare the efficacy and safety of naproxen 500 mg twice daily (bd) versus naproxen 500 mg as needed (PRN) for treatment of ankle sprain.	135 patients with ankle sprain presenting at the emergency department of a teaching hospital. Mean ages were 29.8 ± 10.7 years and 34.08 ± 15.07 years and gender distribution was 40 (64.5%) and 33 (55%) for male in the study arms.	Block randomized, parallel group trial	Appropriateness of prescription and administration	Adherence to therapeutic regimen was higher in the bd arm but there was no difference in analgesic effectiveness.	High to moderate
						Physical effects	Overall pain reduction was unaffected by mode of prescription.	High
						Adverse events	PRN regimens appeared safer than the twice daily regime, possibly due to a significant lower consumption in the number of tablets.	High to moderate
Comparison of the efficacy and safety of once-daily dosing and on-demand use of udenafil for type 2 diabetic patients with erectile dysfunction [32]	2015	South Korea	To compare the efficacy and safety of once-daily dosing with on-demand use of udenafil for type 2 diabetic patients with erectile dysfunction (ED).	141 patients with type 2 diabetes in seven healthcare centres.The sample was all male with the mean ages of 54.44 ± 6.00 years and 53.88 ± 6.07 years in the study arms.	Randomized, open-label, parallel-group	Physical effects	No differences in efficacy or biomarkers of endothelial function were identified.	High
						Adverse events	No significant difference was found between once-daily and PRN dosing with regard to treatment-related adverse drug reactions.	High to moderate

* **Population:** Older people, patients, healthcare providers. **Interventions:** PRN drug use and comparison with other types of drug use patterns.

**Table 2:** Characteristics of excluded studies.

Title	Authors	Year	Country	Aim	Methods	Findings	Reason for Exclusion
The traditional method of oral as-needed pain medication delivery compared to an oral patient-controlled analgesia device following total knee arthroplasty [33]	Lambert, T.L., Cata, D.M.	2014	USA	To compare postoperative pain control afforded by usual care—PRN oral pain medication—with the new oral patient-controlled analgesia device.	Quantitative, survey of thirty patients in each group.	The device offered a significant improvement: less pain, less interference with general activity, mood, sleep and appetite during the first 2 post-operative days and 24 h before discharge.	Survey
As required versus fixed schedule analgesic administration for postoperative pain in children [34]	Hobson, A., Wiffen, P.J., Conlon, J.A.	2015	UK	To assess the efficacy of PRN versus fixed schedule analgesia administration for the management of postoperative pain in children under the age of 16 years.	Systematic review, three RCTs of 246 children aged lower than 16 years.	No conclusions were drawn, due to limited evidence.	Systematic review
Patient controlled opioid analgesia versus non-patient controlled opioid analgesia for postoperative pain [35]	McNicol, E.D., Ferguson, M.C., Hudcova, J.	2015	USA	To assess efficiency and safety of PCA in comparison with non-patient controlled analgesia of PRN for relieving postoperative pain.	Meta-analysis, 1725 participants in the PCA group and 1687 participants in the non-patient controlled group.	PCA was associated with significantly: lower pain scores on visual analogue scales (VAS), greater satisfaction with opioids, higher consumption of opioids and higher incidence of pruritus.	Meta-analysis
Patient controlled opioid analgesia versus conventional opioid analgesia for postoperative pain [36]	Hudcova, J., McNicol, E., Quah, C., Lau, J., Carr, D.B.	2006	USA	To investigate the efficiency of PCA in comparison with conventional analgesia for controlling postoperative pain.	Meta-analysis, 2023 participants in the PCA group and 1838 participants in the non-patient controlled control group.	PCA afforded better pain control and patient satisfaction than conventional opioid analgesia.	Meta-analysis
The effects of as-needed nalmefene on patient-reported outcomes and quality of life in relation to a reduction in alcohol consumption in alcohol-dependent patients [37]	François, C., Rahhali, N., Chalem, Y., Sørensen, P., Luquiens, A., Aubin, H.J.	2015	France	To evaluate the effect of as-needed nalmefene vs. placebo on health-related quality of life (HRQoL) in patients with alcohol dependence.	Quantitative, *post hoc* subgroup analysis of 2 RCTs with 667 patients.	The majority of patients with as-needed nalmefene had significant improvements in HRQoL drinking behaviour and total alcohol consumption.	All arms received a preparation PRN. Comparisons were between nalmefene and placebo.
Systematic review of the predisposing, enabling and reinforcing factors which influence nursing administration of opioids in the postoperative period [38]	Yin, H.H., Tse, M.M., Wong, F.K.	2015	China	To describe factors affecting nurses’ decision-making related to PRN administration of opioid analgesics for postoperative pain.	Systematic review of 39 qualitative and quantitative studies.	Nurses’ knowledge of pain management and opioid analgesia was the main perceived barrier to administration of effective pain relief.	Systematic review
A randomized clinical trial of the efficacy of a self-care intervention to improve cancer pain management [39]	Rustøen, T., Valeberg, B.T., Kolstad, E., Wist, E., Paul, S., Miaskowski, C.	2014	Norway	To assess the efficacy of the PRO-SELF Pain Control Program on pain control and opioid intake in comparison with usual care among out-patients with bony metastases.	Quantitative, a clinical trial of self-care, 87 participants in the PRO-SELF group and 92 participants in the control group.	Both groups reported significant reductions in pain intensity scores and in hours per day in pain. Total opioid consumption increased over time in both groups.	No focus on PRN.
Post-operative pain: the impact of prescribing patterns on nurses’ administration of analgesia [40]	Simons, J., Moseley, L.	2008	UK	To measure the difference between prescribed analgesia and administered analgesia in children during the first 24 h after surgery.	Quantitative, a retrospective chart review of 175 children.	Less paracetamol was administered when prescription was on a PRN basis.	Retrospective cohort
*Pro re nata* (as needed) medication in nursing homes: the longer you stay, the more you get? [41]	Dörks, M., Schmiemann, G., Hoffmann, F.	2016	Germany	To examine predictors of PRN administration in nursing homes.	Quantitative, cross-sectional review of medicines charts of 852 residents in 21 homes.	Most (74.9%) residents were treated with at least one PRN medication. Acetaminophen (paracetamol) was the drug most commonly administered PRN, prescribed to 33.9% residents. PRN prescription was predicted by duration of residence and polypharmacy.	A cross sectional chart review
Pain medication in German nursing homes: a whole lot of metamizole [42]	Hoffmann, F., Schmiemann, G.	2016	Germany	To assess the use of analgesics, particularly metamizole (not available in UK) in nursing homes.	Quantitative, cross-sectional review of medicines charts of 852 residents in 21 homes.	More than half the residents received at least one analgesic. The most frequently prescribed medications were metamizole and paracetamol, the latter as PRN. The proportion of residents receiving metamizole increased with age. Patient safety concerns were raised by the authors.	A cross sectional, retrospective, chart review
Examining trends in the administration of “as needed” medications to inpatients with behavioural and psychological symptoms of dementia [43]	Neumann, R.D., Faris, P., Klassen, R.	2015	Canada	To identify trends in the administration of PRN medications to inpatients with dementia.	Quantitative, retrospective review of medicines charts, 170 inpatients with dementia in neurology wards.	Younger patients received more PRN prescriptions. PRN prescriptions were more common following evening shift change or during weekends. Where patients were receiving regularly scheduled medication from the same drug class, there was a risk of double dosing, exceeding dosage guidelines.	Retrospective chart review
Effect of hospice nonprofessional caregiver barriers to pain management on adherence to analgesic administration recommendations and patient outcomes [44]	Mayahara, M., Foreman, M.D., Wilbur, J., Paice, J.A, Fogg, L.F.	2015	USA	To assess hospice nonprofessional caregivers’ adherence to analgesic administrations and patient outcomes.	Quantitative, a short-term longitudinal correlational study of 46 patient–caregiver dyads.	Higher caregiver adherence to PRN analgesic regimens was associated with lower patient pain intensity and higher patient quality of life.	A longitudinal study
Behavioural and psychological symptoms of dementia: how long does every behaviour last and are particular behaviours associated with PRN antipsychotic agent use? [45]	Voyer, P., McCusker, J., Cole, M.G., Monette, J., Champoux, N., Ciampi, A., Belzile, E., Richard, H.	2014	USA	To assess the course of behavioural and psychological symptoms of dementia (BPSD) over a period of 6 months.	Quantitative, a secondary analysis of a prospective observational cohort study of 146 nursing home residents from 7 homes.	PRN administration of antipsychotic medication was associated with nocturnal BPSD and requesting help unnecessarily. Within 3 months, most BPSD were resolved by usual care and use of PRN antipsychotic medication was not associated with behaviours that put the residents or their caregivers at risk.	Prospective cohort
PRN prescribing in psychiatric inpatients: potential for pharmacokinetic drug interactions [46]	Davies, S.J., Lennard, M.S., Ghahramani, P., Pratt, P., Robertson, A., Potokar, J.	2007	UK	To assess the prevalence of PRN regimens and the potential interactions involving PRN medications in mental health wards.	Quantitative, a cross-sectional survey of prescription charts of 323 inpatients.	In 2089, 48% of prescription items were on a PRN basis. One fifth of patients were prescribed drug combinations interacting via CYP2D6 or CYP3A4, with potential for clinical harm. This included one or more drugs prescribed on a PRN basis.	A cross-sectional review of medicines charts
Administration of PRN medications and use of non-pharmacologic interventions in acute geropsychiatric settings: implications for practice [24]	Lindsey, P.L., Buckwalter, K.C.	2012	USA	To evaluate the effect of PRN psychotropic medications and non-pharmacological interventions to manage psychological symptoms in older adults.	Quantitative, a retrospective chart audit of 108 medical records for patients ≥ 55 years or older admitted to two inpatient geropsychiatric units over a 3-month period.	Insufficient documentation was found regarding PRN administrations and non-pharmacological interventions to identify the best clinical practice.	A retrospective chart audit and review
Nurses’ opinions on appropriate administration of PRN range opioid analgesic orders for acute pain [23]	Gordon, D.B., Pellino, T.A., Higgins, G.A., Pasero, C., Murphy-Ende, K.	2008	USA	To investigate nurses’ opinions of the appropriate implementation of dose-range orders.	Quantitative, online survey of 602 nurses in a medical centre.	Nurses who attended pain management courses were more likely to respond appropriately to questions on patient management than those who did not.	A cross-sectional survey
A study of the prescription and administration of sedative PRN medication to older adults at a secure hospital [16]	Haw, C., Wolstencroft, L.	2014	UK	To investigate the risks of polypharmacy, high dose medications and adverse drug reactions to sedative PRN medications.	Quantitative, review of patients’ records of 92 older adults and 242 working age patients.	Lorazepam was the most commonly administered PRN drug and violence was the most common reason for administrating it. Documentation of adverse drug reactions and patient outcomes was considered suboptimal. Older people received less PRN medication and lower doses.	A retrospective record review
*Pro re nata* medication for psychiatric inpatients: time to act [21]	Hilton, M.F., Whiteford, H.A.	2008	Australia	To evaluate PRN administration of psychotropic medications in term of mental health policies, professional ethics and PRN administration protocols.	Literature review	Development of best practice guidelines is an essential need for the use of PRN administration.	Literature review

**Table 3:** The Cochrane Risk of Bias Assessment.

Author (Year)	Selection Bias		Performance Bias	Detection Bias	Attrition Bias	Reporting Bias	Other Bias
	Random Sequence Generation	Allocation Concealment	Blinding of Participants and Personnel	Blinding of Outcome Assessment	Incomplete Outcome Data	Selective Reporting	
Chibnall et al. (2005) [29]	Unclear	Unclear	Low	Low	Low, 2 of 25 participants did not complete the RCT	Low	Unclear, very small sample size, 1 care home, support from the manufacturer of the medicine investigated.
Baker et al. (2008) [15]	High	High	High, no attempt to blind	Unclear	Unclear, no information	Moderate, no information on adverse effects	High
Morad et al. (2012) [30]	Low	Unclear	High, no information	High-patients and recovery staff were not blinded.	Unclear, 6/34 and 9/34 were excluded for Protocol violations.	High, per protocol not intention to treat analysis	Unclear, very small sample size, single centre
Hajimaghsoudi et al. (2013) [31]	Low	Unclear, no information	High, open label	High, open label	Unclear, no information	Unclear, missing data due to patient non-compliance	Unclear
Park et al. (2015) [32]	Unclear	Unclear	High, open label	Unclear	Moderate, 10/80 participants lost in each arm. No reasons given.	Low, all adverse events were reported	Unclear
